# Gaussian smoothing and modified histogram normalization methods to improve neural-biomarker interpretations for dyslexia classification mechanism

**DOI:** 10.1371/journal.pone.0245579

**Published:** 2021-02-25

**Authors:** Opeyemi Lateef Usman, Ravie Chandren Muniyandi, Khairuddin Omar, Mazlyfarina Mohamad

**Affiliations:** 1 Faculty of Information Science and Technology, Research Centre for Cyber Security, Universiti Kebangsaan Malaysia, Bangi, Selangor, Malaysia; 2 Department of Computer Science, Tai Solarin University of Education, Ijebu-Ode, Ogun State, Nigeria; 3 Faculty of Information Science and Technology, Research Centre for Artificial Intelligence Technology, Universiti Kebangsaan Malaysia, Bangi, Selangor, Malaysia; 4 Faculty of Health Sciences, Center for Diagnostic, Therapeutic and Investigative Studies, Universiti Kebangsaan Malaysia, Jalan Raja Muda Abdul Aziz, Kuala Lumpur, Malaysia; University of Engineering & Technology, Taxila, PAKISTAN

## Abstract

Achieving biologically interpretable neural-biomarkers and features from neuroimaging datasets is a challenging task in an MRI-based dyslexia study. This challenge becomes more pronounced when the needed MRI datasets are collected from multiple heterogeneous sources with inconsistent scanner settings. This study presents a method of improving the biological interpretation of dyslexia’s neural-biomarkers from MRI datasets sourced from publicly available open databases. The proposed system utilized a modified histogram normalization (MHN) method to improve dyslexia neural-biomarker interpretations by mapping the pixels’ intensities of low-quality input neuroimages to range between the low-intensity region of interest (*ROI*_low_) and high-intensity region of interest (*ROI*_high_) of the high-quality image. This was achieved after initial image smoothing using the Gaussian filter method with an isotropic kernel of size 4mm. The performance of the proposed smoothing and normalization methods was evaluated based on three image post-processing experiments: ROI segmentation, gray matter (GM) tissues volume estimations, and deep learning (DL) classifications using Computational Anatomy Toolbox (CAT12) and pre-trained models in a MATLAB working environment. The three experiments were preceded by some pre-processing tasks such as image resizing, labelling, patching, and non-rigid registration. Our results showed that the best smoothing was achieved at a scale value, *σ* = 1.25 with a 0.9% increment in the peak-signal-to-noise ratio (PSNR). Results from the three image post-processing experiments confirmed the efficacy of the proposed methods. Evidence emanating from our analysis showed that using the proposed MHN and Gaussian smoothing methods can improve comparability of image features and neural-biomarkers of dyslexia with a statistically significantly high disc similarity coefficient (DSC) index, low mean square error (MSE), and improved tissue volume estimations. After 10 repeated 10-fold cross-validation, the highest accuracy achieved by DL models is 94.7% at a 95% confidence interval (CI) level. Finally, our finding confirmed that the proposed MHN method significantly outperformed the normalization method of the state-of-the-art histogram matching.

## Introduction

Magnetic resonance imaging (MRI) has been commonly used as a very simple non-invasive imaging technique to study and examine human brain anatomy in order to explain the neuropathogenic causes of various learning disorders, including dyslexia [1–5]. With the increasing generation of MRI data of various phonological and cognitive subsystems of human brains, the chances of machine learning (ML) methods and, more recently, deep learning (DL) methods for dyslexia diagnosis, is promising. However, the achievement of state-of-the-art high accuracy, sensitivity, and specificity for ML and DL methods for dyslexia prediction depends largely on biological interpretability of the sub-anatomical structure of different brain tissue features found in the input MRI dataset. Such features are otherwise referred to as neural-biomarkers and constitute the neuroimaging dataset’s key region of interest (ROI). Lack of biological interpretability is consequent upon variations in the scanner-dependent acquisition methods and protocols as well as differences in the timeframe between MRI data acquisition for the same subject [6], hence the need for image smoothing and intensity normalization.

Although lacking intensity normalized MRI do not have any direct impact on clinical diagnosis of developmental dyslexia by medical doctors or radiologists, the situation can be complicated by some image pre-processing and post-processing tasks that precede ML analysis. These tasks include ROI segmentation, quantitative tissue analysis (estimation of tissue volumes), image registration, and classification, which are highly dependent on intensity information to achieve efficient performance [7–9]. In addition, the majority of ML-based neuroimaging studies for dyslexia neural-biomarkers discrimination require the use of vast amounts of multi-site MRI datasets to evaluate the anatomical variations and alterations in the brains of the study participants. Such datasets are scanned using various scanner types with inconsistent parameter settings at different geographical locations within and across subject classes, as seen in the studies by Plonski et al. [10,11] and Jednorog et al. [12]. While the use of a multi-site MRI dataset provides a way of examining a greater number of subjects to develop a unique dyslexia diagnostic cohorts [11], the method is sometimes hindered by noise and high-intensity variations with a significant negative impact on the results of ML classifiers [13]. The existence of traditional MRI units, by implication, makes direct quantitative analysis difficult. Specifically, MRI datasets are acquired in arbitrary units which are not comparable between study visits within a single subject or between different subjects or groups [9]. For the above reasons, the smoothing and intensity normalization algorithms are expected to improve the distribution of pixels or voxels for each MRI dataset to match the consistency of the chosen high-quality baseline scan, otherwise referred to as the reference image. This will help in enhancing image homogeneity and comparability of essential tissues, more specifically the grey matter (GM), white matter (WM), and cerebrospinal fluid (CSF) between different MRI scans [14]. Therefore, the intensity normalization and smoothing aim to correct scanner-dependent variations for accurate interpretation of the relevant tissues and neural-biomarkers [8].

In this study, Gaussian smoothing and modified histogram normalization (MHN) methods are proposed in order to achieve homogeneous image intensity for brain MRIs. The MRI dataset generated under different field strengths and acquisition parameters scanners, was collected from two publicly accessible databases to analyze inherent dyslexia neural-biomarkers. It is however, hypothesized that proposed smoothing and normalization methods would improve the image pre-processing tasks that enhance the performance of proposed DL models for dyslexia neural-biomarkers discrimination. The proposed smoothing and normalization method’s performance was evaluated based on three image analysis experiments: ROI segmentation, GM volume estimation, and DL classification. The objective of our study is, therefore, to demonstrate how MHN and Gaussian smoothing can improve the biological interpretations and quantification of GM volumes in the ROIs, and thus, improve the classification accuracy of dyslexia neural-biomarkers prediction. The contributions of this study are summarized thus:

- Design a novel mechanism for improving biological interpretations and homogeneity of dyslexia neural-biomarkers inherent in multi-site MRI datasets.
- Efficient segmentation of GM brain tissue with a statistically high mean DSC index of 0.7756 and low MSE of about 8.1153.
- Statistically significantly high tissue volume estimations for segmented ROIs at *p*-value<0.05 and;
- Improved DL classification accuracy of 94.7%, the sensitivity of 95.8%, the specificity of 94.9%, and F-Score of 95.4% at significantly low feature extraction time of 12.7 minutes.

## Related works

Several previous studies have highlighted the significance of intensity normalization and smoothing with some various proposed methods focusing on medical image processing. Previous studies such as Nyul and Udapa [15] have suggested a normalization method consisting of two key stages: training and transformation. The standard image histogram scale parameters are determined at the training stage while the candidate volume histograms are mapped to the standard histogram scale at the transformation stage. This method was validated and improved upon by Shah et al. [16] and Nyul et al. [6] in their various independent studies. Collewet et al. [17] have investigated the impacts of normalization on different acquisition protocols in a texture classification study for old and young soft cheeses. Meier and Guttmann [14] suggested an intra-scan and inter-scan normalization approach for serial MRI scans for direct quantitative analysis. Gonzalez & Woods [18] proposed a histogram mapping method, while Koptenko [19] has implemented a contrast stretch normalization method based on the maximum and minimum grayscale values in the image. Christensen [20] proposed a histogram even-order derivative analysis normalization method for brain MRI scans with a good WM peak distinction, regardless of the similarity between GM and WM peaks. Wang et al. [21] proposed a histogram matching method to correct variations due to the scanner’s sensitivity in the brain MRI analysis. The method achieved a 5% variation reduction in WM intensity for all images of the study participants.

In order to correct low-reliability landmark tissue representation that characterized Nyul’s algorithm [15], Madabhushi and Udupa [22] implemented two-scale concepts proposed in Madabhushi et al. [23]. This method improves tumor segmentation for multiple sclerosis (MS) using an MRI dataset. Arguably, existing normalization methods suffer from limited biological interpretability of the normalized space and are considerably slower due to the strong reliance on non-rigid registration to suit the histogram. The results of post-processing tasks, such as ROI segmentation and classification, are, therefore, of poor reliability and low accuracy. However, to enhance MRI intensity normalization, Sun et al. [8] proposed a simple histogram normalization method. In this method, the intensity of a low-quality input image was made to range between the minimum and the maximum intensity values of the high-quality image through stretching and shifting its histogram to cover all the available grayscale levels. Furthermore, many methods of neuroimaging data smoothing exist. Among them are wavelet filter [24], fourth-order partial differential equation [25], total-variation-norm denoising scheme [26,27] and template-based filtering procedure [28]. Awate and Whitaker [29] proposed nonparametric neighbourhood statistics for the MRI dataset smoothing based on the Rician noise model. Basu et al. [30] proposed a Perona-Malik-like noise filter that assumes a known noise level for the Rician noise model in combination with a local Rician data attachment term. Martin-Fernandez et al. [31] proposed the Wiener-filter technique, while Aja-Fernández et al. [26] proposed a method of smoothing MRI dataset using the Rician noise estimator for linear minimum mean square error (LMMSE). The Rician noise model assumes zero-mean uncorrelated Gaussian noise with equal variance in the MRI dataset, hence the use of Gaussian smoothing proposed in this study. Gaussian smoothing is computationally powerful in the sense that, since it is a linear low-pass rotationally symmetric filter, it gives weight to higher significance pixels near the edge with the ability to control the degree of smoothness.

## Materials and methods

**Fig 1:**
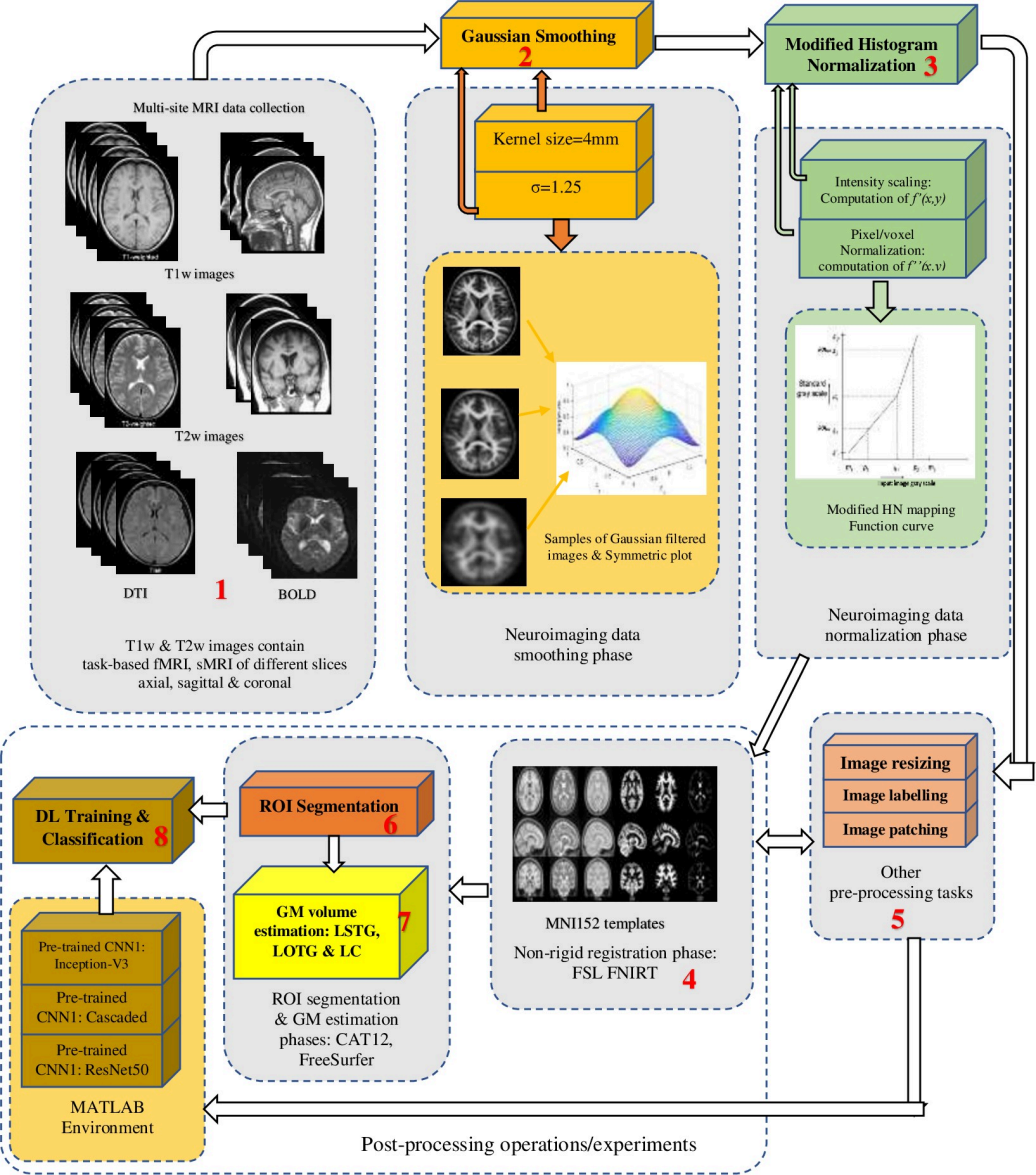
Dyslexia neural-biomarkers interpretability and classification mechanism. In the above mechanism, multi-sites neuroimaging datasets are smooth using a Gaussian filter followed by normalization using a modified histogram method, template registration, ROI segmentation, GM estimations, and DL classification. Other pre-processing steps are required during DL training and classification.

The mechanism for improving dyslexia neural-biomarker interpretations and DL classification is presented in Fig 1. As shown in the figure, the entire mechanism comprises 8 distinct modules. The first module is multi-site/multi-parameters MRI data acquisitions followed by some image pre-processing operations include labelling, uniform resizing, noise removal, otherwise known as smoothing. The proposed Gaussian smoothing and MHN methods are contained in module 2 and 3 respectively. Post-processing operations used to evaluate the smoothing and normalization performance include ROI segmentation, GM volume estimation, as captured in modules 6 and 7, and finally, DL classification shown in module 8. For DL classification experiments, the entire images were split into 64×64 small, randomly overlapping patches using a patching algorithm. They were resized accordingly to create an augmented dataset, which addresses the effect of overfitting on the proposed pre-trained CNN models. The image normalization and smoothing are essential operations because of the variations in the acquisition protocols, electromagnetic field noise, scanner field strength, and different acquisition parameters. The detail about the proposed mechanism is described in the subsequent sub-sections. All post-processing operations are linked with other pre-processing operations of module 5.

### Module 1 and 2: MRI data acquisition and pre-processing

The MRI datasets used in the analysis were obtained from two publicly available databases. At first, a total of 97 randomly selected brain MRI scans were collected from the pool of available datasets on the *OpenNeuro* repository via https://openneuro.org. The datasets have been stored according to the specifications of the Brain Imaging Data Structure version 1.3.0 [32] and were collected from 91 schoolchildren between 8.7 and 15.5 years of age (mean age = 11.4, SD = 2.1, 39 females). All brain MRI scans were performed using a 1.5T General Electric Signal Excite scanner at Evanston Hospital, IL, USA, from recruited participants in the greater Chicago area. Participants were recruited through advertisements, community events/organizations, and brochures. The datasets comprise two types of MRI scans obtained but different acquisition protocols: structural MRI (sMRI) and task-based functional MRI (fMRI). The following acquisition parameters were used for the collection of all sMRI scans. These scans comprises: T1-weighted spoiled gradient recalled echo (SPGR) images, repetition time (TR) = 33.333ms, echo time (TE) = 8ms, acquisition matrix (AM) = 256×256, bandwidth = 114.922Hz/Px, slice thickness (ST) = 1.2mm, number of slices (NoS) = 124, voxel scale (vs) = 1mm isotropic, flip angle (FA) = 30 deg. Blood oxygen level-dependent signal (BOLD) images were obtained for all fMRI scans type. These scans comprise T2-weighted susceptibility single-shot echo-planar imaging (EPI). Acquisition parameters are as follows: TR = 2000ms, TE = 25ms, AM = 64×64, bandwidth = 7812.5Hz/Px, ST = 5mm, NoS = 24, vs = 3.75×3.75×5mm, FA = 90 deg. Generated slices were acquired in layers from bottom to top, in an odd-first arrangement manner. According to the datasets’ authors, the datasets were originally collected to analyze neural and lexical developmental processes underlying children’s auditory and visual modalities in rhyming, spelling, and semantic decision tasks. Therefore, the images of subjects showing impaired visually, auditory, and phonology (reading/spelling) for the given tasks during task-based scanning are categorized as dyslexic. The details about participant demographics, lexical processing tasks, reading, cognitive tests, participants selection criteria, etc. can be found in Lytle et al. [33].

Secondly, 25 randomly selected brain MRI samples were collected from the Connectivity-based Brain Imaging Research Database (C-BIRD) of Beijing Normal University (BNU). These images were used to validate the proposed smoothing and normalization methods and also for training the proposed DL models. According to the information contained in the above database, which was accessed via http://dx.doi.org/10.15387/fcp_indi.corr.bnu1, there are two types of resting-state fMRI scans (i.e., T1-w and T2-w) and DTI datasets, all scanned at different acquisition parameters. These datasets were obtained from 57 healthy young volunteers (aged 19–30 years) at two different time intervals. Every volunteer completed two separate MRI scan sessions at an interval of approximately 6 weeks (40.94±4.51 days). All participants were right-handed without a history of any neurological or mental illness. The acquisitions which have been approved by the State Key Laboratory of Cognitive Neuroscience and Learning at BNU Institutional Review Board, have their parameters summarized thus: fMRI scans were obtained using a 3T Siemens Avanto TrioTim Scanner with a 12-channel head coil for 3D MPRAGE [34] whole-brain scans (144 saggital slices for anatomical, 33 axial slices for resting-state). The AM = 256×192, TR = 2530ms, TE = 3.39ms, FA = 7 deg, FOV = 256mm, and acquisition time (AT) = 8.07ms. Acquisition parameters for the second type of resting-state fMRI scans include AM = 64×64, TR = 2000ms, TE = 30ms, FA = 90 deg, FOV = 200mm, and AT = 6.46ms. DTI scans were obtained using the same scanner for 62 slices with AM = 128×128, TR = 8000ms, TE = 89ms, FOV = 282mm, and AT = 8.07ms. None of the participants was diagnosed with impaired vision and hearing or any other serious neurological disorders, such as ADHD and Alzheimer’s disease.

**Fig 2:**
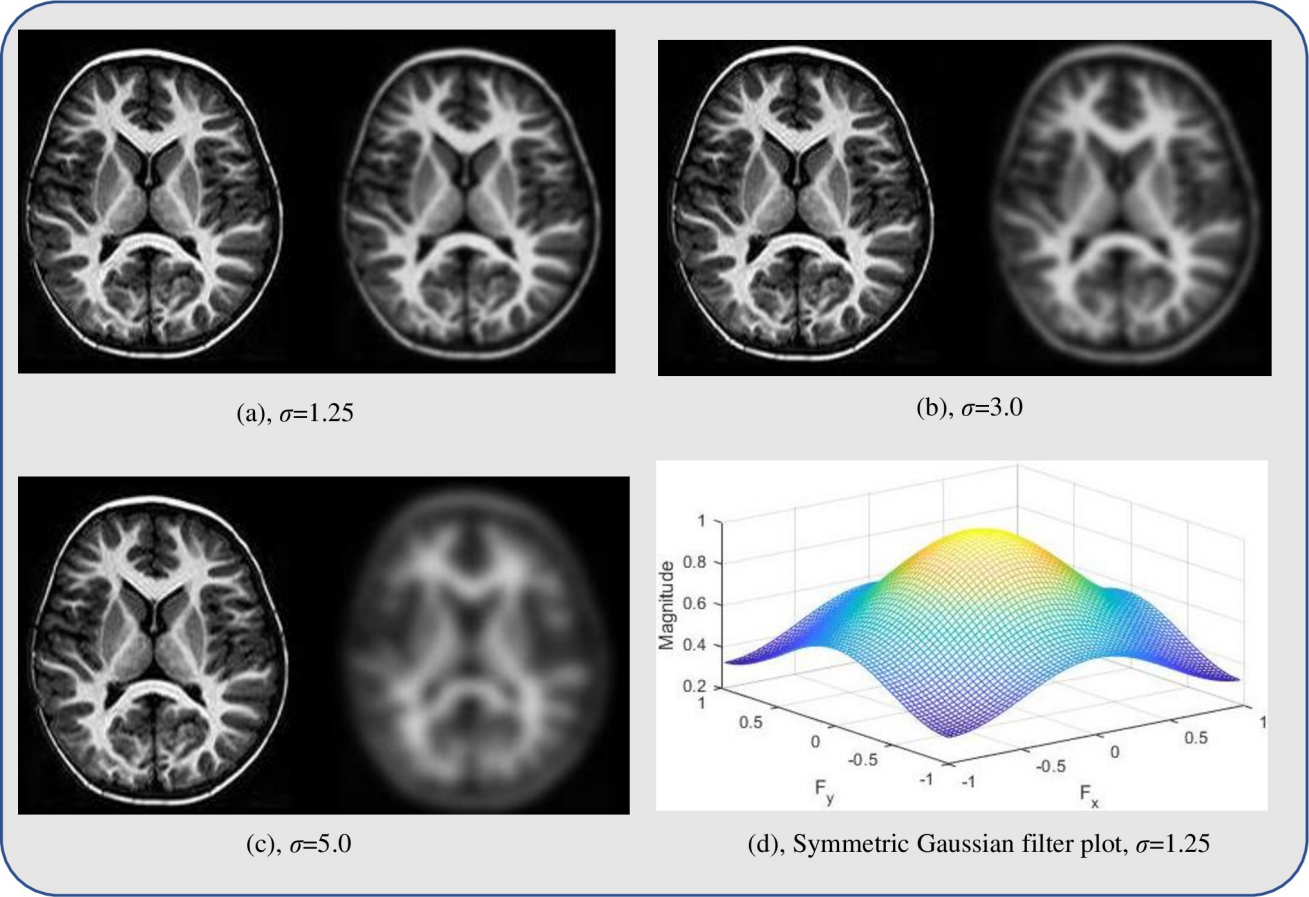
Visual comparison of a neuroimage sample at a different value of *σ*. In (a), (b), and (c), the left side is a noisy image; the right side is a Gaussian smooth image. (a) GM area which constitutes the main ROI of the study is distinguishable from WM and background areas after smoothing; for (b) GM is fairly distinguishable after smoothing; (c) GM area becomes blur when *σ*>3; (d) shows the symmetrical property of the filter at *σ* = 1.25.

**Fig 3:**
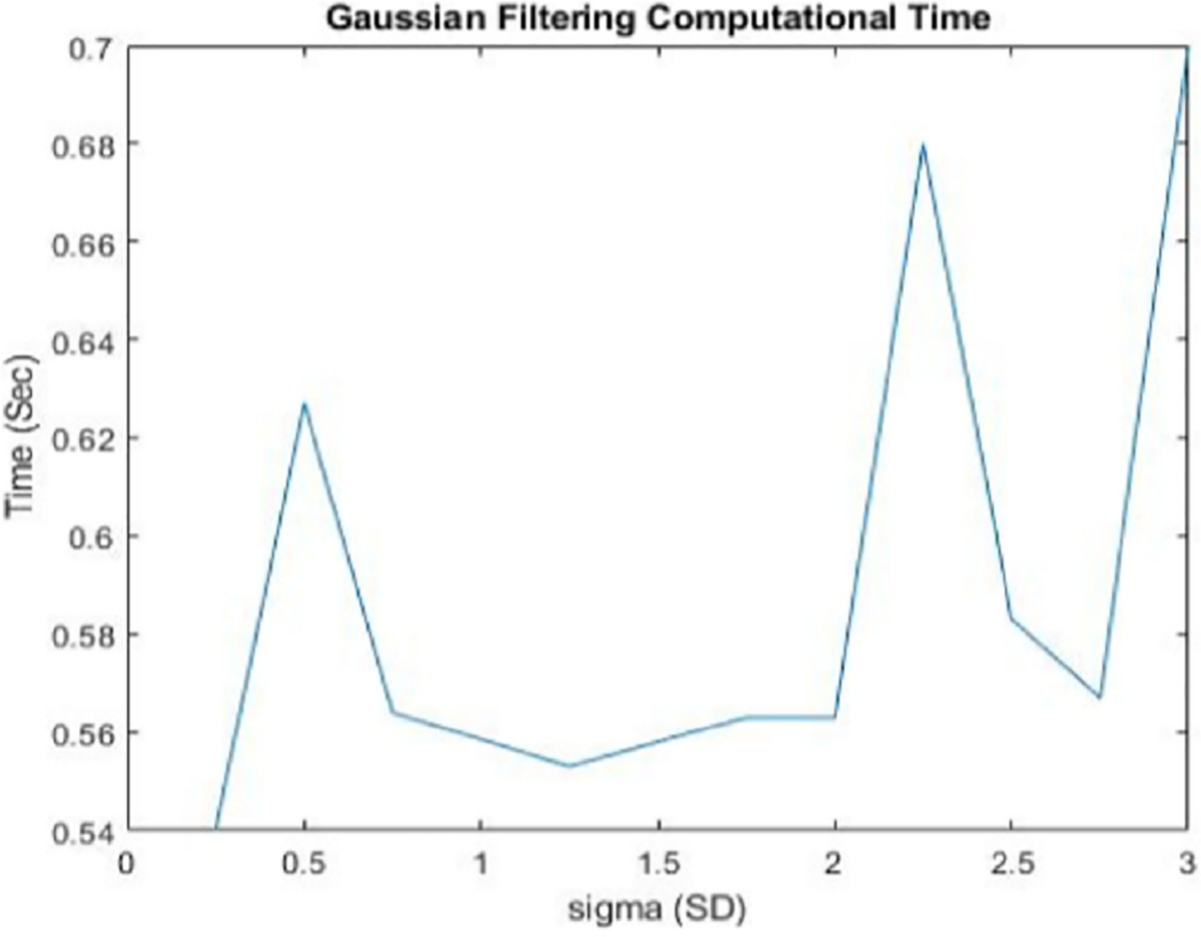
Time-behaviour of Gaussian filter at a different scale, *σ* values on a neuroimage data sample. This shows that all smoothing process requires less than 1 second to complete regardless of the value of *σ*. Sigma (SD) axis represents *σ* values.

All datasets were first accessed using ITK-SNAP software package [35] and were saved as MetaImage file formats with (.raw) extension. They were, after that, read in the MATLAB program and saved as jpeg formats. Smoothing of all images was achieved using a Gaussian isotropic kernel of size 4mm (Gaussian filter) at a scale value, *σ* ranges between [0.5, 3.0], as shown in module 2 of Fig 1. The third module is the implementation of the proposed MHN method. Implementation of the above modules improves image quality by removing unnecessary details such as noise, variations, and impractical detail from textual image output, thus, enhancing the readability of neural-biomarkers of anatomical information contained in the dataset [36]. After initial resizing, the best smoothing performance was achieved at a scale value, *σ* = 1.25 at execution time, *t* = 0.563 seconds on a sample of neuroimage data before applying the same space value to smooth other neuroimaging datasets used. The peak signal-to-noise ratio (PSNR) between the reference image and the Gaussian smooth image was 18.21 after denoising. There is a 0.9% improvement in the denoised image compared to the PSNR value obtained prior to smoothing. Fig 2 shows the visual comparison of a sample of neuroimage at different *σ* values, while Fig 3 graphically illustrate the behaviour of the Gaussian filter at these values relative to their execution times. It can be deduced from Fig 3 that all smoothing tasks were completed in less than 1 seconds regardless of the value *σ*.

### Module 3: Proposed modified histogram normalization (MHN) method

By definition, normalization algorithms transform each neuroimage scan’s distributions to the chosen standard image quality baseline to achieve homogeneity and promote the comparability of images between different MRI scans [7,8,14]. In an attempt to select the most effective normalization method for brain lesions dataset acquired through intra-scan and inter-scan MRI machines, Loizou et al. [7] compared six different normalization algorithms. They found that histogram normalization (HN) allows accurate computation of texture features that distinguish between normal and lesion tissues. Furthermore, Sun et al. [8] found that the HN normalized intra-scan MRI dataset improves image registration accuracy, tissue-types segmentation, tissue quantification, and brain template construction. Consequent upon the above justifications, we modified the existing HN method to suit patterns and features present in the study neuroimaging datasets.

In the proposed MHN method, two steps are involved, namely: intensity scaling and normalization. Assuming that the minimum and maximum intensity levels on the standard scale are denoted by *q*_*min*_ and *q*_*max*_, firstly, during intensity scaling, the low-intensity region of interest (*ROI*_*low*_) and the high-intensity region of interest (*ROI*_*high*_) in the original (reference high-quality) image are identified from its histogram after smoothing. The intensities of the image are mapped to the values between *ROI*_*high*_ and *RO*I_low_ using a function *g(x*,*y)* shown in Eq (1):

$$g(x,y)=\frac{f(x,y)-{ROI}_{low}}{{ROI}_{high}-{ROI}_{low}}$$ (1)

where *f(x*,*y)* is a grayscale value of the original image at coordinate *x* and *y* and *g(x*,*y)* is the corresponding transformed greyscale value. Secondly, during the normalization, the original image is stretched and shifted to cover all the grayscale levels in the image using a function *h(x*,*y)* shown in Eq (2):

$$h(x,y)=\frac{{ROI}_{high}-{ROI}_{low}}{q_{max}-q_{min}}(g(x,y)-q_{min})+{ROI}_{low}$$ (2)

If the target histogram of the original image, function *g*(*x*,*y*) starts at *q*_min_ and spreads up to *q*_max_ grayscale levels in the intensity region of interest (ROI), then the image can be scaled up between the lower boundary *m*_1_’ and the upper boundary *m*_2_’ so that the pixels in the normalized image *h*(*x*,*y*) lie between the minimum intensity level (*ROI*_low_) and the maximum intensity level (*ROI*_high_). The variables *m*_1_ and *m*_2_ represent the lower boundary and the upper boundary of the original image before scaling.

**Fig 4:**
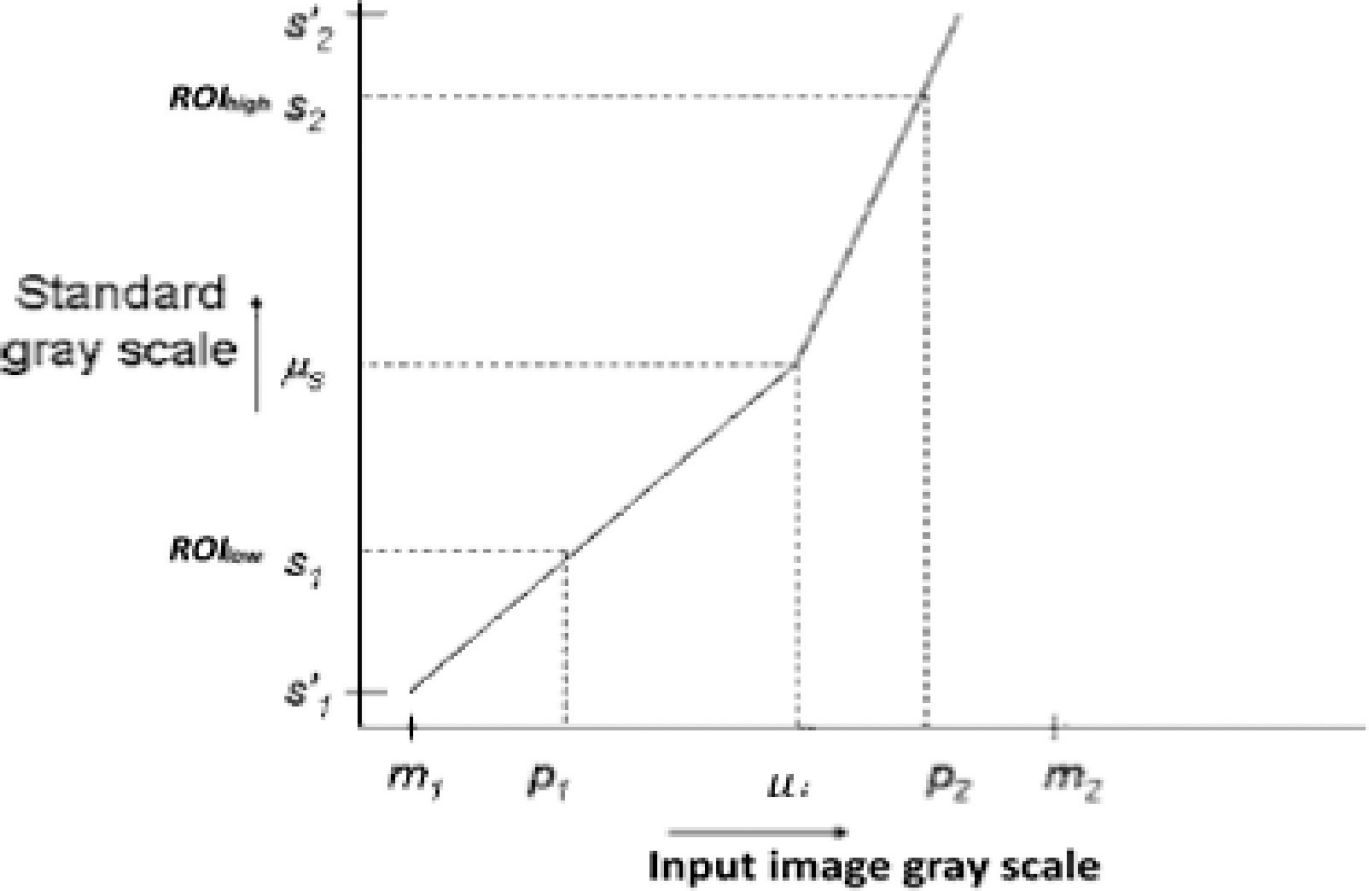
Mapping function curve for MHN. The first mapping is from [*P*_*1*_, *μ_i_*] to [*S*_*1*_, *μ_s_*] and the second mapping is from [*μ_i_*, *P*_*2*_] to [*μ_s_*, *S*_*2*_]. The lower and the upper boundaries of the standard scale are *m1’* and *m2’*, respectively.

The above image normalization can be accomplished by creating two distinct linear mappings. The first mapping is [*P*_*1*_,*μ*_i_] to [*S*_*1*_,*μ*_*s*_] and the second mapping is [*μ*_*i*_,*P*_*2*_] to [*μ*_*s*_,*S*_*2*_] as shown in the mapping function curve displayed in Fig 4. Subsequently, the lower and upper ends of the standard scale are applied to *S′*_*1*_ and *S′*_*2*_, respectively, by mapping [*m*_*1*_,*P*_*1*_] to [*S′*_*1*_,*S*_*1*_] and [*S’2*,*m*_*2*_] to [*S*_*2*_,*S′*_*2*_]. This mapping is called the normalization of the input image from the intensities [*S′*_*1*_,*S′*_*2*_] to [*m*_*1*_,*m*_*2*_] of the standard scale. The normalization function is defined in Eq (3) as *N(x*,*y)*. Note that, for simplicity purpose, *S*_*1*_ = *ROI*_*low*,_ and *S*_*2*_ = *ROI*_*high*,_ respectively, in Fig 4.

$$N(x,y)=\left\lceil \begin{array}{c} \mu_{s}+(g(x,y)-\mu_{i})\frac{S_{1}-\mu_{s}}{P_{1}-\mu_{i}}\\ \mu +(g(x,y)-\mu )\frac{S_{2}-\mu_{s}}{P_{2}-\mu_{i}} \end{array}\right\rceil ,\;m_{1}^{\prime }\leq g(x,y)\leq \mu_{i}\leq m_{2}^{\prime }$$ (3)

where ⌈∎⌉ denote the ’*ceiling*’ operator, *μ_i_* and *μ_s_* are the mean values for the input image histogram and original histogram, respectively. *P*_*1*_ and *P*_*2*_ are pixel values from the input image. In this study, three important intensity values were considered contrary to the approach suggested by Nyul et al. [6]. The purpose is to avoid unreliable dependent on landmarks in the histogram for normalizing used neuroimaging dataset. These intensity values include minimum, maximum, and mean value, indicating that the proposed normalization method is different from the popular histogram matching normalization (HMN) method.

**Fig 5:**
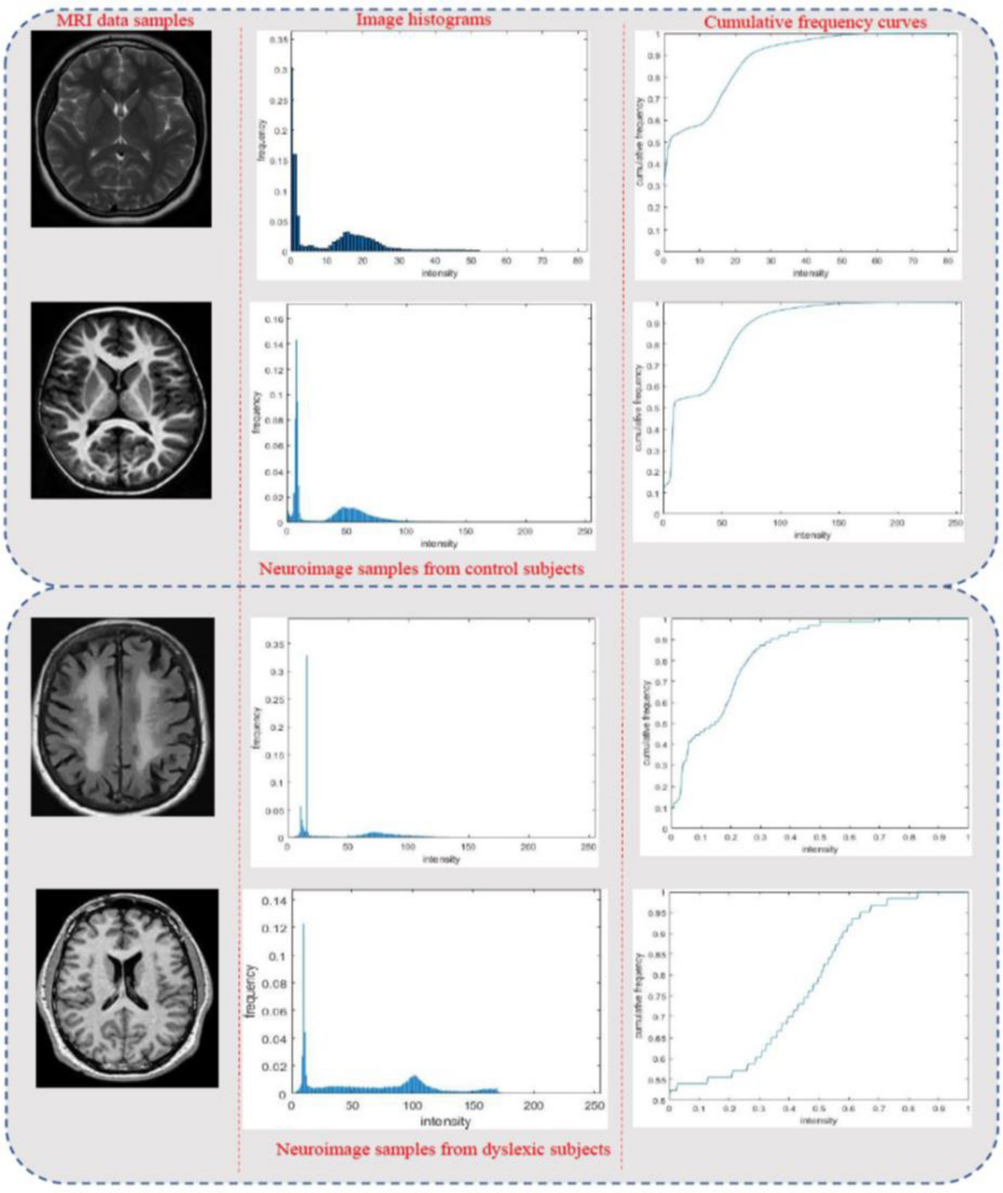
Visual inspection of image histograms and CFCs for four randomly selected neuroimages before MHN. (a) the first column shows image samples from both controls and dyslexic group, (b) the second column shows their transformed histograms, while (c) the third column shows their CFCs. The first two rows are samples drawn from the control group, while the last two rows are samples drawn from the dyslexic group.

In this study, it was found that the proportions of intensity levels for the same type of tissue for the neuroimages of subjects within the same group from the same scanners were similar despite variations in field strengths and acquisition parameters. Prior to normalization, visually examination of histograms and cumulative frequency curves (CFC) from randomly selected neuroimages across the two subject groups (dyslexics and controls) and across different scanner types was performed. As shown in Fig 5, there are wide variations in the grayscale intensity levels of all the selected images. The high-quality (reference) image for each group was then used to normalize the low-quality images (input images) between the *ROI*_low_-*ROI*_high_ ranges of its intensity. The procedure is repeated across groups to have uniform intensity normalization for all images. The proposed smoothing and normalization algorithms’ performance was evaluated to analyze improvements to the accuracy and reliability of three post-processing neuroimaging datasets analysis. In each scenario, results from MHN normalized dataset were then compared against the most popular HMN method. These post-processing tasks include tissue segmentation, ROI gray matter volume estimation, and DL classifications, described in the subsequent sections. From the histograms and CFCs of Fig 5, it can be deduced that image pixels are not evenly distributed across the entire greyscale levels in most of the samples shown prior to histogram normalization.

### Module 4, 5 and 6: ROI segmentation

Image segmentation is a critical step in many applications involving image analysis, video processing, and computer vision in general. It is also used to divide the image into separate regions that ideally correspond to specific objects in the real world, especially in medical image processing [37,38]. Segmenting neuroimages into different tissue types, specifically GM, WM, and CSF, is a vital post-processing activity and significantly affects the efficiency of other post-processing operations, such as tissue volume estimation, feature extraction, and neural-biomarkers detection. In this study, segmentation of neuroimaging dataset into three main brain tissues was accomplished using Markov random field segmentation algorithm [39] implemented on computational anatomy toolbox version 12 (CAT12). This software package is an extension of statistical parametric mapping (SPM12) and implementable in a MATLAB environment. At first, a non-rigid image registration into Montreal Neurological Institute (MNI152) brain template [40] was performed on the reference image, and all the MHN normalized images using FSL FNIRT software tool [41]. After that, cognitive/phonological features relating to GM, WM, and CSF tissues were retrieved with a focus on only three brain regions. These regions are otherwise referred to as ROIs of the study respectively. They include the left superior temporal gyrus (LSTG), left occipital temporal gyrus (LOTG), and lateral cerebellum (LC). Comparative evaluation of the segmentation results is conducted by measuring a spatial overlapping index between the images using the dice similarity coefficients (DSC) defined in Eq (4) as:

$$DSC(X,Y)=\frac{2|X\cap Y|}{\left|X|+|Y\right|}$$ (4)

where *X* and *Y* represent cardinalities of binary labels for two compared segmented images. The DSC index’s value varies from 0 to 1, where 0 indicates no spatial overlap, and 1 indicates total overlap. The mean square error (MSE) value between the MNI152 registered image version and the MHN normalized image was also computed and the results compared against HMN method using Eq (5):

$$MSE=\frac{1}{n}\sum_{i=1}^{n}{\left(\hat{X_{i}}-X_{i}\right)}^{2}$$ (5)

where $\hat{X_{i}}$ is the pixel intensity of the non-rigid registered image, and *X*_*i*_ is the equivalent intensity of the input image.

### Module 7: Gray matter volume estimation

After the segmentation process, the gray matter density in the three-segmented ROIs brain regions was utilized to estimate the textual feature volumes. The purpose of this task is to determine the extent to which the proposed MHN and smoothing methods have improved the biological interpretability of the texture feature contained in the MRI dataset used. To accomplish this task, FreeSurfer software tool downloaded at http://surfer.nmr.mgh.harvard.edu/ was used to count the GM volumes in the three brain regions, i.e., LSTG, LOTG, and LC for all the subjects before and after normalization. Volume difference between groups and across the same subject class for both dyslexics and controls were then compared. This procedure was validated by manual measurement conducted by an experienced radiologist and fMRI expert at UKM Teaching Hospital, Malaysia. According to studies by Plonski et al. [10,11], FreeSurfer morphometric measurements exhibits good test reliability outputs across different scanner manufacturers and across multiple field strengths. The GM volumes before and after normalization between the input and referenced images for the same subject class are finally compared with an additional comparison with the HMN method.

### Module 8: Deep learning models, training and classification procedure

This section presents the details of how the proposed pre-trained CNN models were used to discriminate dyslexia neural-biomarkers from normal (control) brain features using normalized and smoothen neuroimaging datasets. Each neuroimage data was randomly patched to generate more features, which helps to deal with the overfitting problem. In order to extract the deep features from the patched images, three state-of-the-art depth-based pre-trained networks were implemented. Their training process follows a transfer learning paradigm with parameter fine-tuning from the top *n*-block layers. In our case, the proposed models have been adapted by carefully updating weights using our task-specific training data patches. The value of *n* = 4,3,5, respectively, for each of the three selected models. As shown in module 8 of Fig 1, these models include Inception-V3, two-ways cascaded CNN, and ResNet-50. The choice of these models was based on the premise that CNN models with increased depth can better approximate the target function to the number of non-linear mappings, resulting in better feature representations as demonstrated by the previous studies [42–44].

#### Implementation of Inception-V3 model

**Fig 6:**
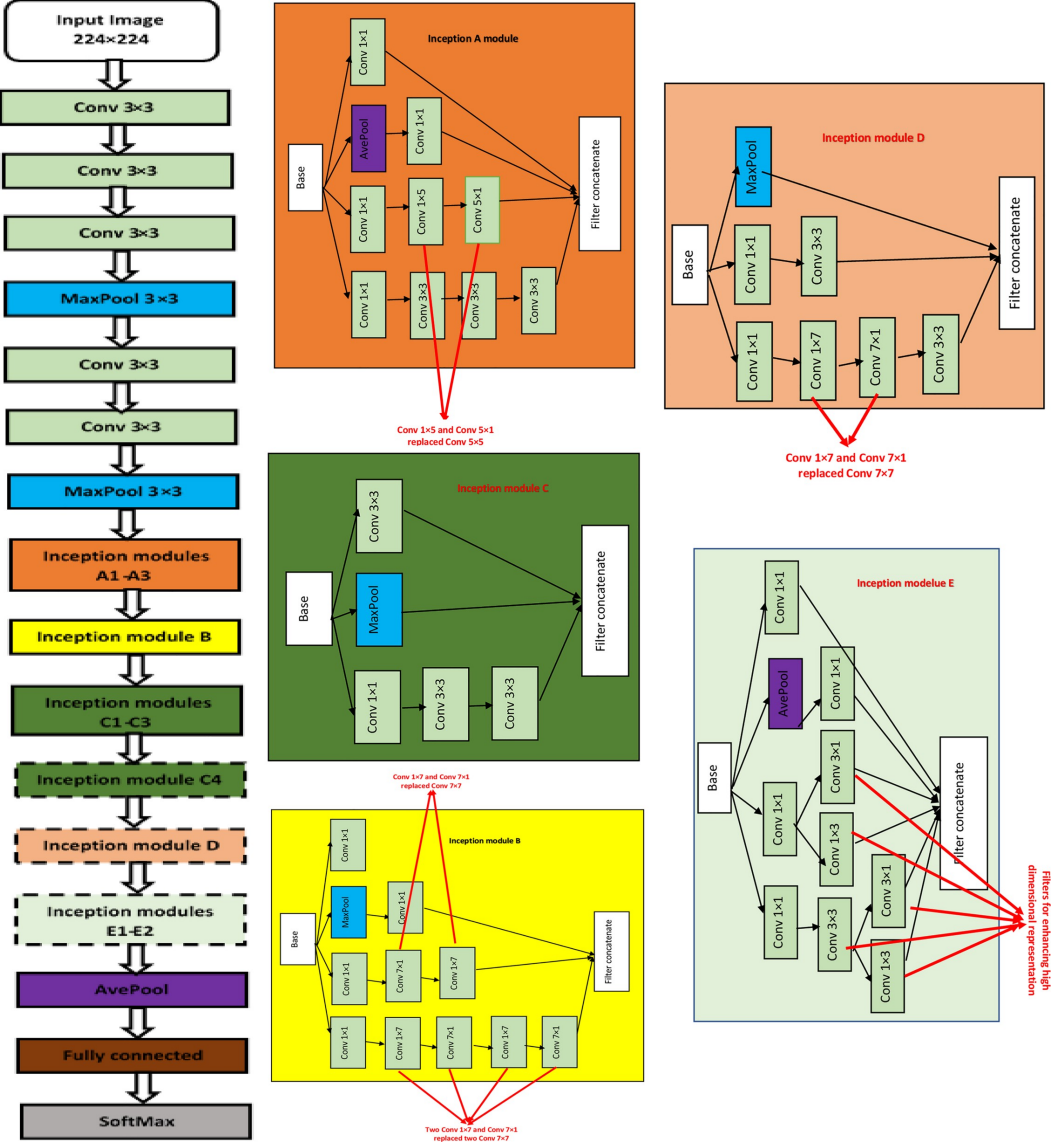
Inception-V3 architecture. Blocks in dotted lines represent modules that can be removed in this experiment. (a) is the Inception-V3 model, (b)-(f) are architectures of Inception modules A-E.

In the first stage of deep models’ implementations, pre-trained Inception-V3 CNN model’s architecture was modified. The modified model maintains a depth of 42 layers with 11 Inception modules of five different kinds (A-E), as shown in Fig 6. The general goal of training the network is to reduce the computational costs of deeper networks without decreasing its generalization power. This was done by replacing large filters sizes, e.g., 5×5 and 7×7 feature maps with smaller asymmetric filters (1×7 and 1×5) and also, by using 1×1 convolution as a barrier before large filters [45]. Each module consists of a convolution layer, a rectified linear activation unit (ReLU) layer, a pooling layer, and a batch normalization layer. In the proposed model, these modules are concatenated to realize maximum extraction of the input data features. Fig 6(A)–6(E) demonstrate how factorization of 5×5, 7×7 convolutions into smaller convolutions 3×3, or asymmetric convolutions 1×7 and 7×1, is conducted during the experiment. This reduces the number of deep network parameters [46]. Compared against the new classification layers added, the original classification layers replaced include the global average pooling layer and the last dense layer with 1000 outputs. In this case, the new network architecture was able to adapt to neuroimaging datasets for the study.

#### Implementation of Cascaded CNN model

**Fig 7:**
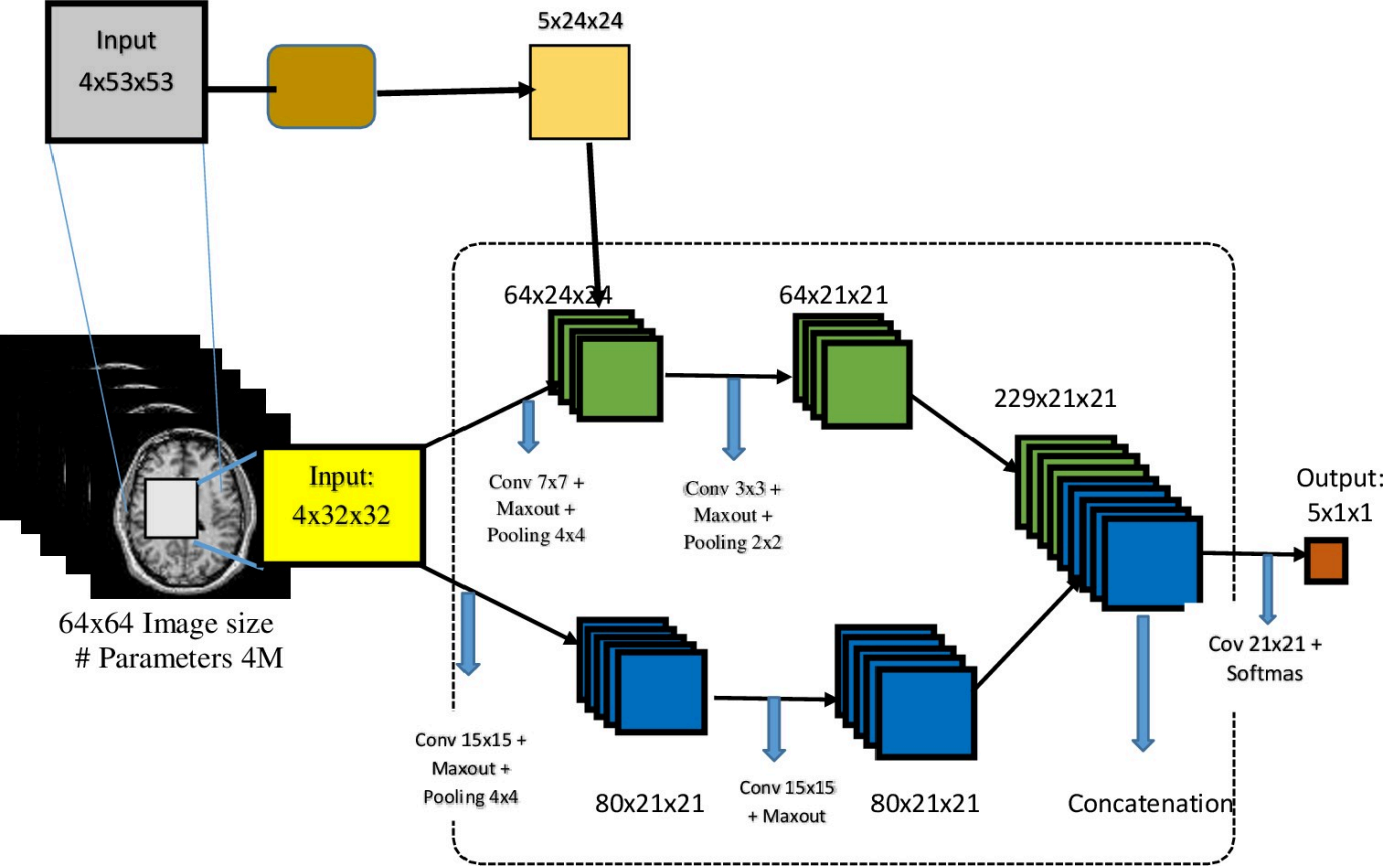
A diagrammatical representation of two-pathways cascaded CNN model.

During the second stage of deep models’ implementations, cascaded CNN architecture was used. Cascaded deep CNN is one of the most recent depth-based CNN architectures that consists of several concatenated CNN models, each predicting a specific aspect of the input image features. The two-pathway cascaded feed-forward CNN model implemented in this study is similar to the one earlier implemented by [47] in both architecture and feature representation manners. As shown in Fig 7, the architecture has two CNN model pathways. The input image goes through two separate pathways of convolution operations. The two pathways were trained concurrently to extract high-level features. The first pathway consists of smaller 7×7 stacked receptive fields, while the second path consists of larger 15×15 stacked receptive fields. The first CNN output line was linked directly to the first hidden layer of the second CNN model, and its outputs were concatenated with softmax activation after each convolution layer. Fully-connected layers were utilized for training and classification, while a dropout layer was included to deal with the risk of overfitting.

#### Implementation of ResNet50 model

**Fig 8:**
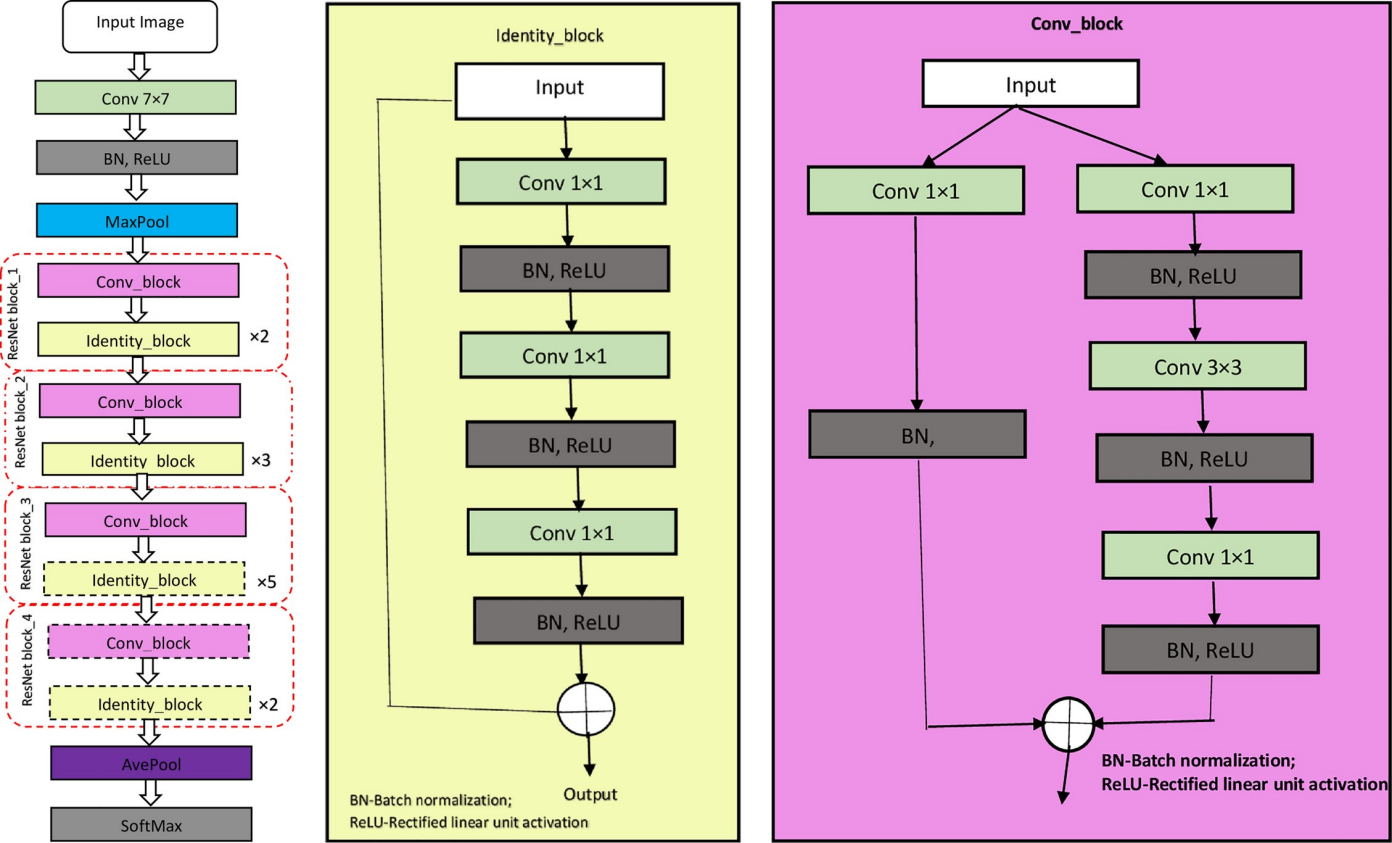
ResNet50 model. ResNet blocks are enclosed in red broken boxes. (a) is the architecture of ResNet50; (b) is the architecture of identity block; (c) is the structure of the convolution block.

In the third and final stage of deep models’ implementations, pre-trained ResNet50 CNN model was used. The proposed model comprises 152 layers, 34 of which are plain, and 34 layers are residual. As shown in Fig 8, there are four stacks of ResNet blocks (enclosed in red broken boxes) in the proposed architecture. Each ResNet block comprises stacked layers of convolution block and repeated identity blocks which represent two kinds of shortcut modules. The identity block does not have a convolutional layer at the shortcut, as shown in Fig 8(B). This makes its input to preserve uniform dimensions as its output. In the case of a convolution block, its input dimensions are smaller than its output dimensions due to the convolutional layer’s availability at a shortcut (Fig 8(C)). In both blocks, 1×1 convolution is implemented at the beginning, and the end of the network through a technique called a bottleneck design [46]. This technique decreases the number of parameters without degrading the performance of the network. In this experiment, some of the deep shortcut modules were replaced with a new set of classification layers specific to study dataset.

#### Training and classification procedure

The training of DL models requires a large volume of data. Compared to natural images, such as ImageNet, which run into several million, our sample size was relatively small. Image patching approach was adopted to reduce input dimensionality, with a transfer learning to obtain effectively trained DL models. The hyper-parameters were then fine-tuned based on the patched images. Small patches are usually more homogeneous than the entire image and can be predicted more precisely [48]. Each image is divided into a series of 50 small randomly overlapping 64×64-pixel patches using some program codes written in MATLAB. Each patch was, thereafter, resized to a 117×117 pixels dimension for training both Inception-V3 and cascaded CNN models and later, to a 224×224 pixels dimension for training the ResNet50 model. Other hyper-parameters of each of the proposed deep models, as adjusted, are summarized in Table 1.

**Table 1:** Parameters settings for DL models utilized in the study. Conv, convolutional layer; fc, fully connected layer; SGD, stochastic gradient descent.

Parameters	Inception-V3	Cascaded model	ResNet50
Input image size	117×117×3	117×117×3	224×224×3
Input kernel size	-	-	-
Number of layers	42	40	152
First conv layer feature maps	55×55×4	53×53×4	55×55×4
First conv layer kernel size	5	3	5
First conv layer stride	3×3	3×3	3×3
Next few conv layer feature maps	24×24×28	21×21×24	27×27×64
No. fully connected layer (fc)	fc800	fc800	fc800
No. of parameters in million	23.2	22.8	25.6
Batch size	10,000 patches	10,000 patches	10,000 patches
Learning rate	0.001	0.001	0.001
Optimizer	SGD	SGD	SGD

**Fig 9:**
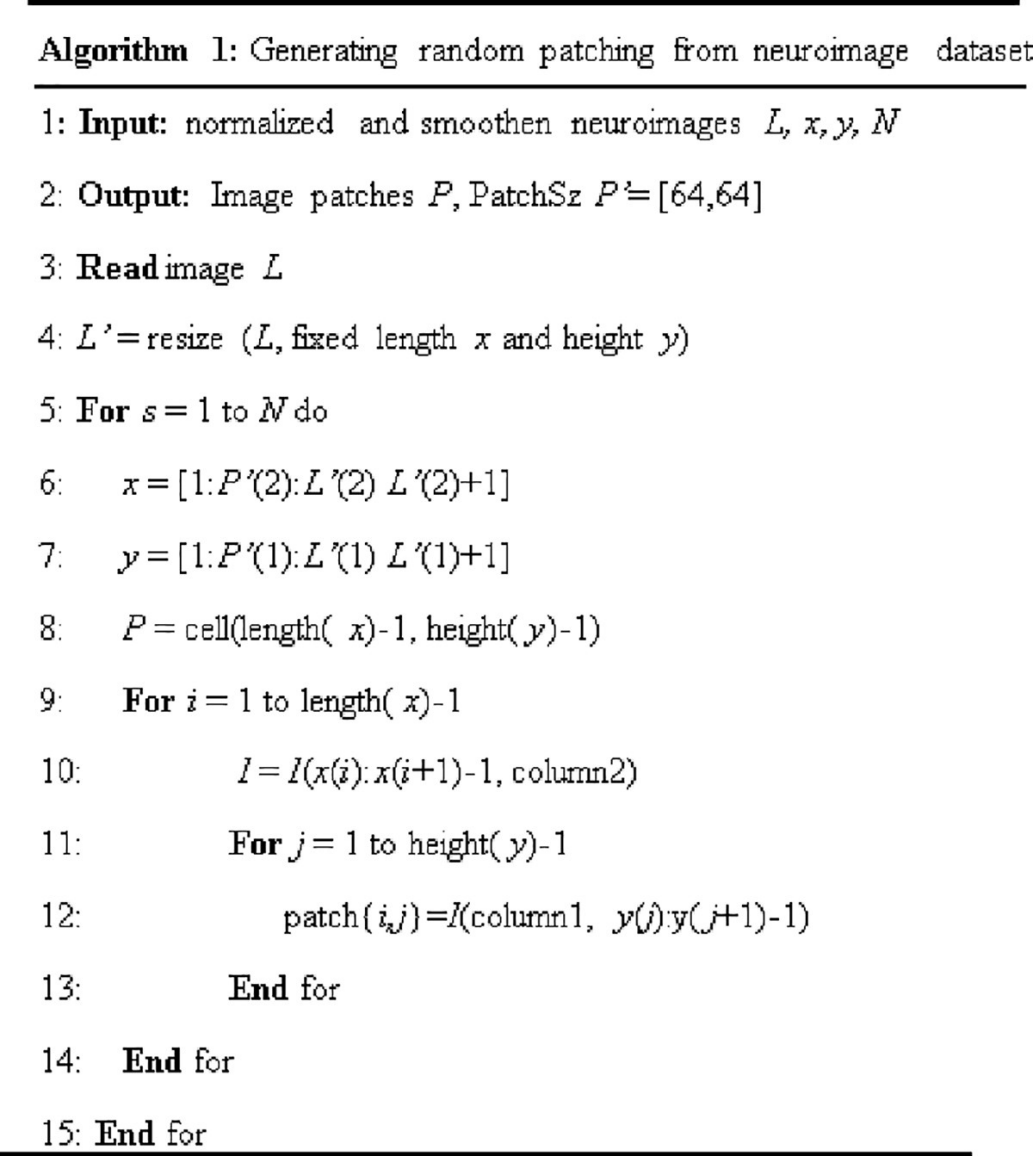
Summary of neuroimaging datasets patching algorithm.

The proposed algorithm for neuroimaging datasets patching is summarized in Fig 9. There was a total of 390,400 image patches after the patching process, including 124,800 patches having dyslexia neural-biomarkers and features, while 265,600 patches have the control (non-dyslexic) features. The evaluation of proposed DL models was based on 10-fold cross-validation (CV) with 70% (273,280 patches) reserved for training and 15% (58,560 patches) each reserved for validation and testing, respectively. In other words, the train-test-validation split ratio of the total image patches is 14:3:3. Binary digit 1 was used to label patches with dyslexia neural-biomarkers, and 0 was used to label features of control patches. The performance of the proposed DL models for dyslexia neural-biomarkers classification were obtained using performance evaluation metrics including accuracy, sensitivity, specificity, and *F*-score, defined in Eqs (6)–(9):

$$Accuracy=\frac{TP+TN}{TP+TN+FP+FN}$$ (6)

$$Sensitivity=\frac{TP}{TP+FN}$$ (7)

$$Specificity=\frac{TN}{TN+FP}$$ (8)

$$F-score=\frac{2\times precision\times recall}{precision+recall}$$ (9)

**Fig 10:**
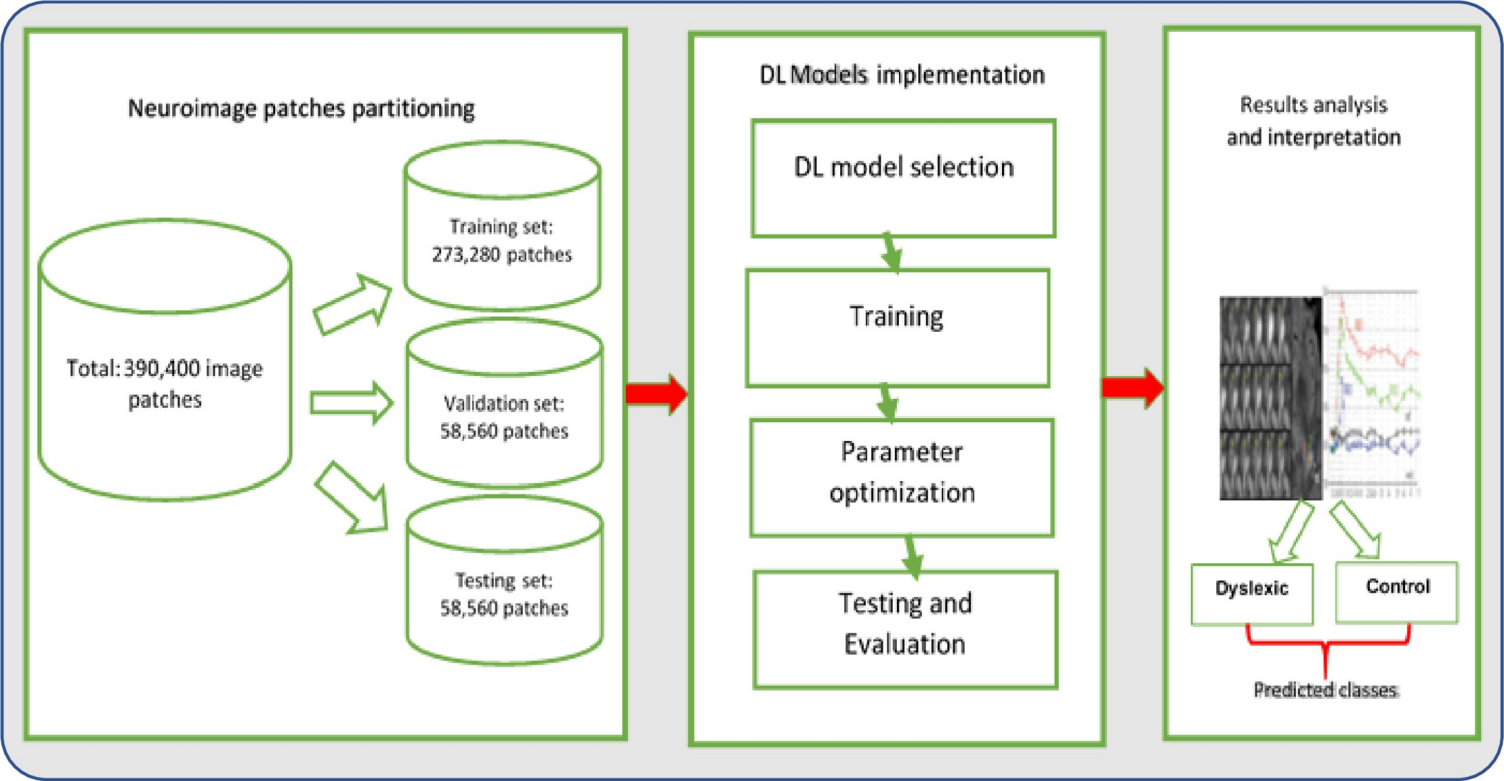
Schematic illustration of training and classification procedure for DL models.

Fig 10 schematically illustrates the procedure for training the proposed DL models. The training and testing of DL models were accomplished at various training iterations with 100 set as minimum training epoch and 550 set as maximum training epoch. The iterations were incremented at an interval of 50 epoch. In-line with Zhao et al. [49], the ’*poly*’ learning rate policy has been used to dynamically update the learning rate for all model convergences where the learning rate was 0.001. The training rate was initialized to 0.1 and steadily decreased by a factor of 10, while the decay value was retained at 0.0005, a momentum of 0.9, and a batch size of 10,000 patches. Stochastic gradient descent (SGD) with momentum algorithm was employed to minimize the negative log-probability for each predicted class. Confidence Interval (CI) levels for prediction accuracy for all three models were estimated to be 95%.

## Experimental setup and results

**Fig 11:**
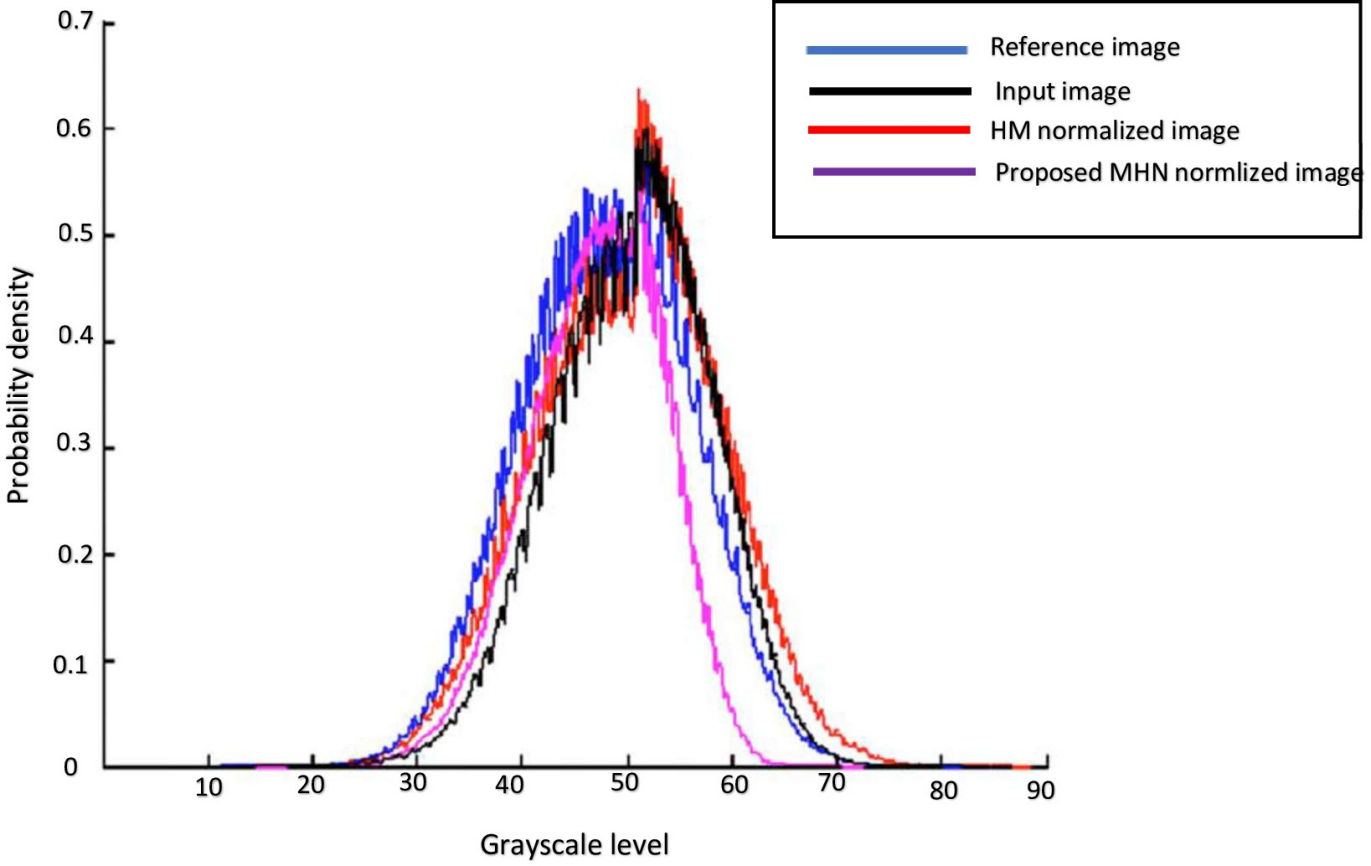
Visualizing the pdf curves of the reference image, input image, and normalized images. HMN-histogram matching normalization; MHN-modified histogram normalization. MHN shows better performance for the input image.

All experiments were performed on a GPU-based system with a 2.70GHz and 8.00GB RAM, and 4 Core (s) Intel (R) processor due to high-speed requirements. The version of the GPU processor on the system used for the experiment is NVIDIA GeForce GTX680. This processor helps to accelerate speedy implementations of DL models. According to [50,51], GPU processors are powerful compared to their CPU-based counterparts in processing speed and memory usage. This is due to their enlarged arithmetic computational capabilities. The performance of the proposed models was evaluated at different levels of training iteration. Table 2 presents the computed mean DSC index of tissue segmentation for all the 97 subjects with and without dyslexia. From this table, it can be observed that the proposed MHN and Gaussian smoothing methods produced statistically significantly high DSC index values for LSTG and LOTG brain regions that were segmented compared to the popular HMN method, whereas the mean DSC index value for LC is comparable for both normalization methods. The degradation in the state-of-the-art HMN method’s performance was because it is highly dependent on several numbers of histogram bins. Visual inspection probability density function (pdf) curves of Fig 11 also shows that the proposed normalization method demonstrates significant improvement over the HMN method, thus improving the biological interpretability of tissue neural-biomarkers and features present in the used neuroimaging dataset. Smoothing of data also significantly enhances the mean DSC index values for all the three cognitive and phonological brain regions analyzed.

**Table 2:** Mean DSC for segmentation of all 97 subjects (dyslexic and control) before and after smoothing and normalization using GM tissue overlaps (mean ± SD).

Segmentation DSC index	LSTG	LOTG	LC	Mean
DSC (Input Image)	0.8124±0.0221	0.7315±0.0436	0.6219±0.0362	0.7321
DSC (normalized image with HMN method [16])	0.8249±0.0197	0.7367±0.0502	0.6424±0.0412	0.7532
DSC (normalized image with proposed MHN method)	0.8561±0.0183*	0.7511±0.0497*	0.6548±0.0338	0.7756*

*Statistically significantly larger than the other two (*p*-value<0.05); SD-standard deviation; LSTG-left superior temporal gyrus; LOTG-left occipital temporal gyrus; LC-lateral cerebellum; HMN-histogram matching normalization; MHN-modified histogram normalization.

As shown in Table 3, the MSE value generated after implementing the proposed smoothing and MHN methods is the lowest compared to the remaining three sets: reference image, the input image, and HMN normalized image. This is observed within and across the two subject groups, i.e., for the dyslexic and control groups. This corroborates Sun et al.’s [8] assertion that the HN method can contribute to many clinical applications through efficient template registration accuracy.

**Table 3:** Summary of MSE within and across the subject groups for dyslexic and control (mean ± SD).

Neuroimage type	Dyslexic	Control	All subject
Reference image	8.1690±0.4186*	8.0702±0.4847*	8.1099±0.4618*
Input image	9.8087±0.4932	10.0228±0.5842	9.9367±0.5593
Normalized with HMN method [16]	8.4431±0.5289	8.4857±0.5564	8.4686±0.5459
Normalized with proposed MHN method	8.1805±0.4178*	8.0714±0.4828*	8.1153±0.4609*

*Statistically significantly lower than the other two; HMN-histogram matching normalization; MHN-modified histogram normalization.

In Table 4, the mean volumes of GM density in the LSTG, LOTG, and LC for normalized input image using the proposed MHN method are closer to the reference image than the result produced by the popular HMN method. Results obtained for GM tissue volumes for the three ROI studied is significantly larger (*p*-value<0.05) after the proposed MHN method than its equivalent HMN method and the input image.

**Table 4:** Mean segmented brain region volume for all 97 subjects (dyslexic and controls) before and after normalization (mean ± SD).

Gray matter volume in selected brain regions	LSTG	LOTG	LC
Volume (reference image)	583.7±61*	411.3±61*	1098.8±133*
Volume (input image)	509.6±63	472.7±28	1082.4±134
Volume (normalized image with HMN method [16])	542.9±65	421.6±28	1077.3±130
Volume (normalized image with proposed MHN method)	581.9±62*	409.1±62*	1093.6±135*

*Statistically significantly larger than the other two (*p*-value<0.05); SD-standard deviation; LSTG-left superior temporal gyrus; LOTG-left occipital temporal gyrus; LC-lateral cerebellum; HMN-histogram matching normalization; MHN-modified histogram normalization.

**Fig 12:**
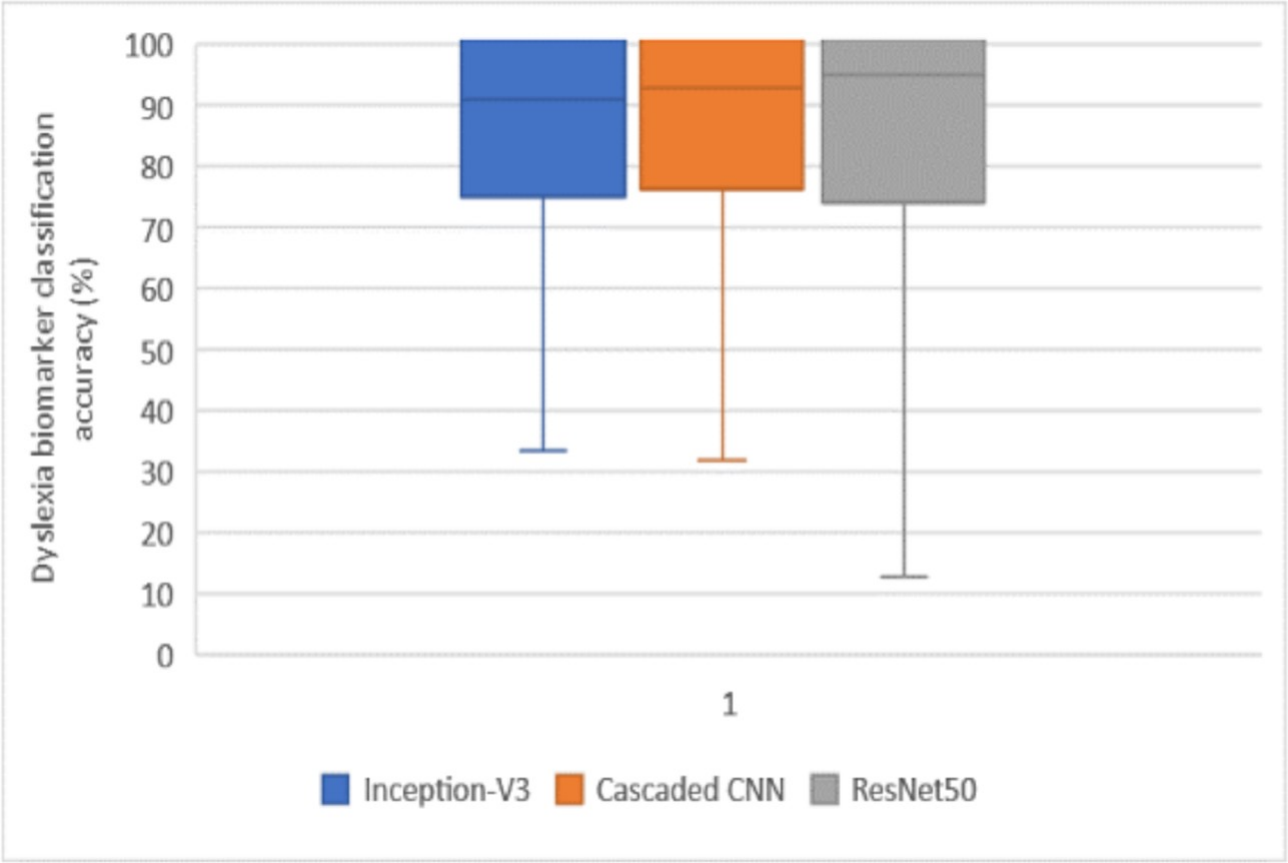
Boxplot showing classification accuracy for the three DL models at a 95% CI level.

The results from the three DL models for dyslexia neural-biomarker discrimination are presented in Table 5 and boxplot of Fig 12. After 10 repeated 10-fold CV for each of the proposed models at a 95% CI level, these results are obtained. All the three models performed significantly high when the datasets have been normalized and smoothen using the proposed methods. Meanwhile, the best performance was observed for ResNet50, which completed feature extraction in a significantly lesser time of 12.65 minutes compared to other models. Although, the three models’ performance is good without smoothing and pre-processing normalization tasks, the models require a longer time to learn high-level abstract patterns of the neural-biomarkers found in the datasets. This is due to the poor comparability and interpretability of important tissue features.

**Table 5:** Performance evaluation of DL models for dyslexia neural-biomarker classification without/with smoothen and normalized dataset (mean ± SD after 10 repeated 10-fold CV).

DL Model		Iteration (Epoch)	Accuracy (%)	Sensitivity (%)	Specificity (%)	F-Score (%)	Feature extraction time (mins)
Inception-V3	Without smoothing and MHN	402	86.23±1.99	88.91±3.78	85.68±3.20	87.27±3.47	41.66
	With smoothing and MHN	380	89.08±1.22	90.22±2.61	92.86±2.14	91.52±2.35	33.45*
Cascaded CNN	Without smoothing and MHN	498	80.91±2.31	91.74±1.88	93.11±3.04	92.42±2.23	44.02
	With smoothing and MHN	550	91.21±0.89	93.11±2.64	92.95±2.46	93.03±2.55	31.78*
ResNet50	Without smoothing and MHN	369	93.33±1.02	95.11±2.87	91.42±0.83	93.23±1.29	23.67
	With smoothing and MHN	450	94.67±0.69*	95.79±2.18*	94.91±2.16*	95.35±2.17*	12.65*

*Statistically significantly larger than the other two (*p*-value<0.05); 95% CI level. MHN-modified histogram normalization; CNN-convolutional neural network.

**Fig 13:**
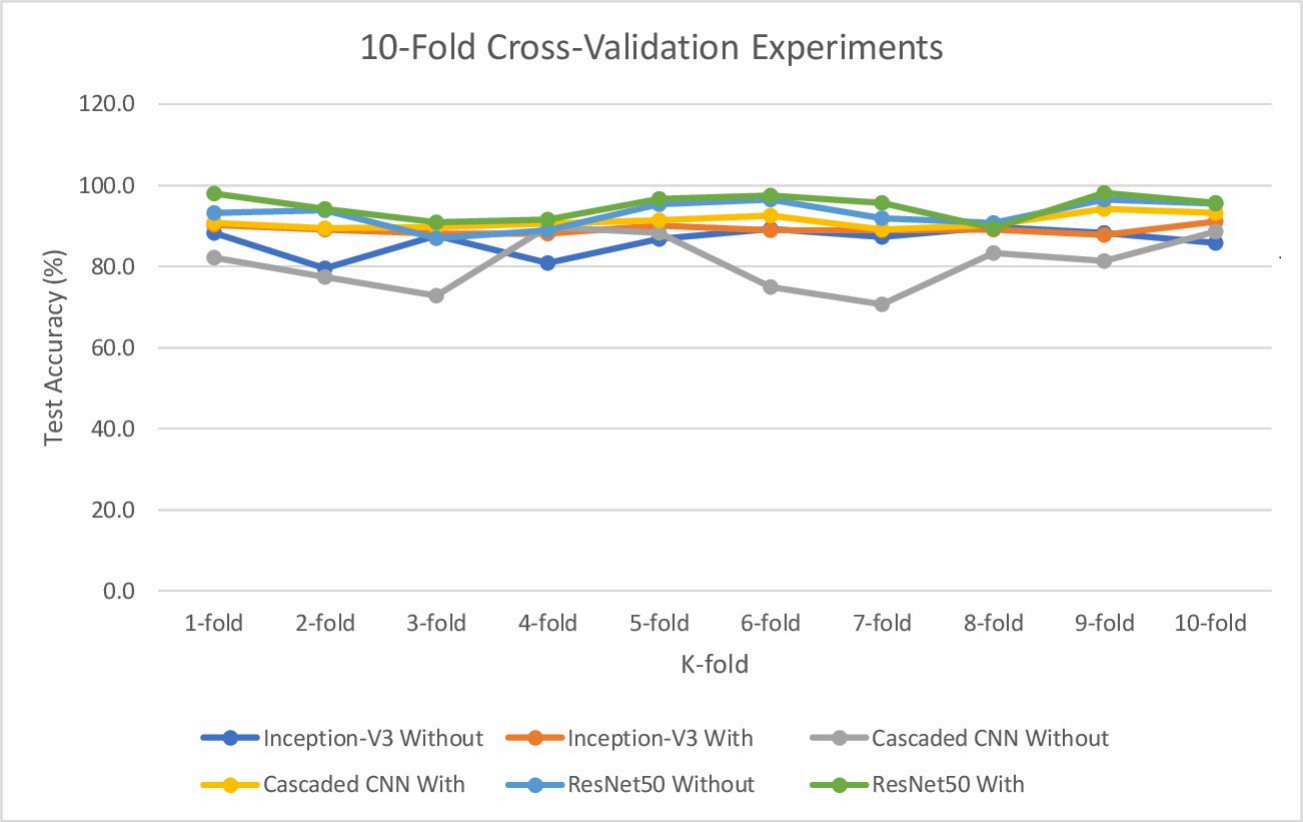
10-fold cross-validation experimental performance for test accuracy.

Table 6 and Fig 13 further illustrate the test accuracy performance behaviours for the proposed DL models. These results were obtained without and then, with implementation of the proposed Gaussian smoothing and MHN methods and confirmed that the performance of the three proposed DL models are significantly improved. The purpose of the test accuracy performance experimental is to validate the earlier results presented in Table 5.

**Table 6:** 10-fold cross-validation experimental performance for test accuracy (%).

Cross-validation k-fold	Inception-V3		Cascaded CNN		ResNet50	
	Without	With	Without	With	Without	With
1-fold	88.2	90.3	82.1	90.7	93.2	97.9
2-fold	79.5	89.1	77.4	89.4	93.8	94.1
3-fold	87.6	88.0	72.8	89.8	86.9	90.8
4-fold	80.8	88.1	89.7	90.6	88.9	91.6
5-fold	86.8	90.1	88.3	91.3	95.4	96.6
6-fold	89.3	88.9	74.9	92.5	96.5	97.4
7-fold	87.2	88.9	70.6	89.1	91.9	95.7
8-fold	89.8	89.0	83.3	90.2	90.7	89.4
9-fold	88.3	87.7	81.4	94.1	96.5	98.1
10-fold	85.7	91.0	88.6	93.2	95.5	95.7
Mean±SD	86.32±3.47	89.11±1.07*	80.91±6.82	91.09±1.67*	92.93±3.29	94.73±3.13*

* Significantly larger than other (p-value<0.05); 95% CI level; SD-standard deviation; Without-without smoothing & MHN; With-with smoothing & MHN; CNN-convolutional neural network.

## Discussion

Findings from the study have shown that intensity normalization and smoothing play a very important role in improving the neuroimaging dataset analysis results for dyslexia neural-biomarkers classification. Such neuroimaging data analysis includes ROIs segmentation, brain tissue density volume estimations, and DL classifications. This is largely due to an improvement in the biological interpretability of neural-biomarkers and features embedded in the neuroimaging datasets.

As shown in Fig 2, the best smoothing output was obtained by Gaussian filter when the value of *σ* = 1.25 at a very reasonable execution time prior to using the same value to smooth entire datasets. The proposed MHN method, combined with the Gaussian smoothing method, can outperform other existing normalization methods for the MRI dataset. This has been demonstrated by comparing the post-processing tasks’ outputs generated by the proposed smoothing and MHN methods with the most popular HMN method.

As presented in Table 2, the proposed MHN and smoothing methods produced statistically significantly high mean DSC index values for the segmented LSTG and LOTG brain regions compared to the HMN method although, the mean DSC index value for LC is comparable for both methods. The implication of the above is that tissue comparability has been significantly improved, resulting in a good biological interpretation of the selected brain regions. Evidence supporting the above findings is shown in Fig 11, where the pdf curve shape of the proposed normalization method is very similar to the reference image used.

The MSE values computed and shown in Table 3 confirmed that the proposed MHN and smoothing methods give the lowest error rates for the input images compared to the popular HMN method. This was observed within and across the two subject groups for the study and corroborates Sun et al.’s [8] claim that the HN method can contribute to many clinical applications, including template registration accuracy.

As illustrated in Table 4, the mean volume estimate for GM tissue density in the LSTG, LOTG, and LC for input image has been improved significantly with the application of the proposed methods. The results obtained for GM volume estimations using the proposed MHN method are closer to the reference image than the result produced by the popular HMN method. The result obtained for GM tissue volume for the three ROI brain areas studied is significantly larger (*p*-value<0.05) after applying the MHN method than its equivalent HMN method for the input image.

**Fig 14:**
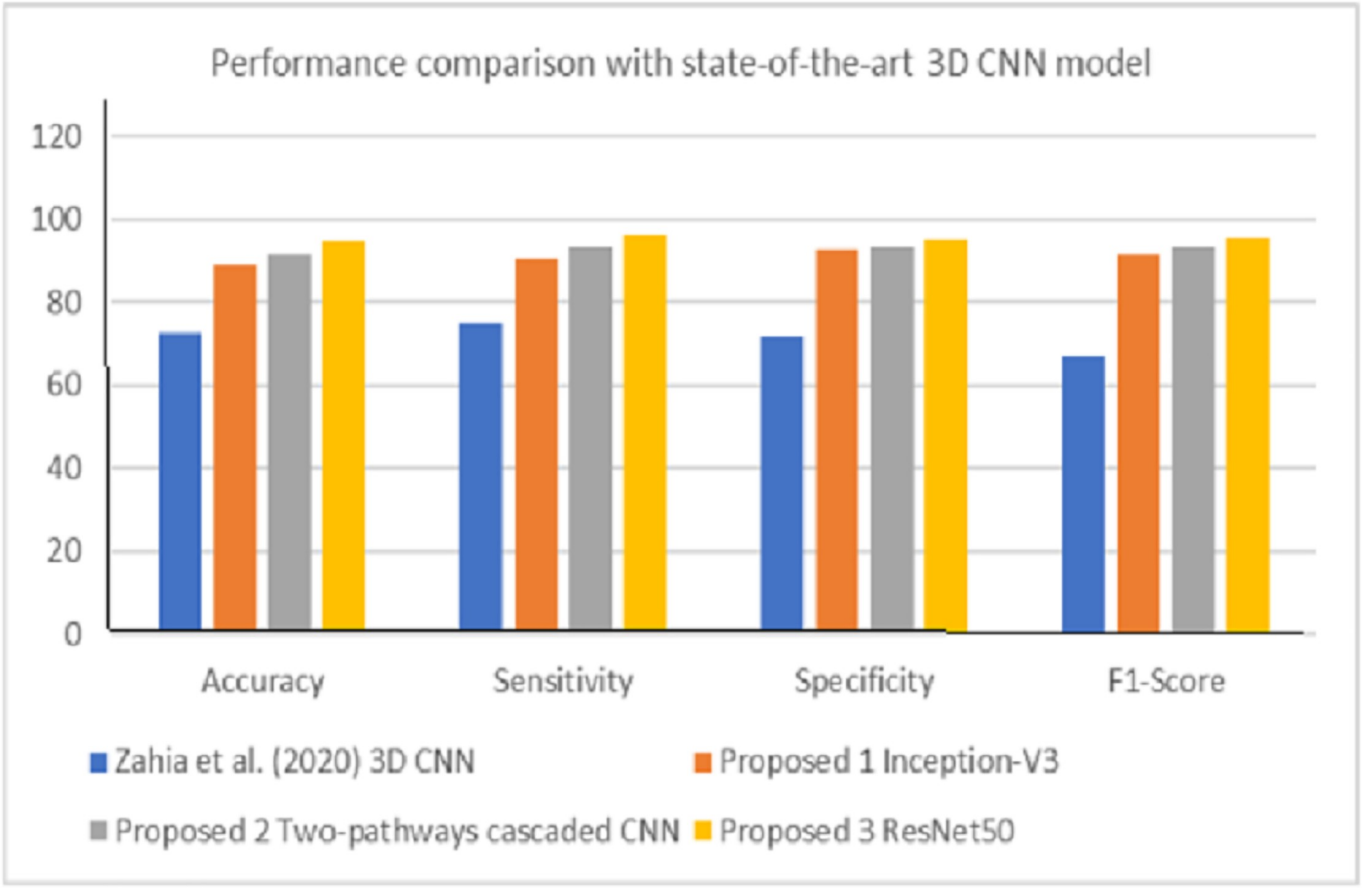
Performance comparison with state-of-the-art DL model for fMRI-based dyslexia study.

Finally, the results presented in Tables 5 and 6 as well as Figs 12 and 13 confirmed the efficiencies of the biological interpretability of features and neural-biomarkers in the neuroimaging datasets used for the study. This can be observed in the high accuracy, sensitivity, specificity, and F-score value returned by the proposed pre-trained DL models. Specifically, the Inception-V3 network generated 89.1% accuracy at 380 epochs at feature extraction time of 33.5 minutes as its best accuracy from the normalized and smoothened datasets. Cascaded CNN produced 91.2% accuracy as its best accuracy at 550 epochs at 31.8 minutes feature extraction time while ResNet50 gives 94.7% accuracy as its best accuracy at 450 epoch and significantly reduced feature extraction time down to 12.7 minutes. The evaluation metrics employed were obtained after 10 repeated 10-fold CV at a 95% CI level. It can be observed that performance of the three models shows significant improvements with normalized and smoothen neuroimaging datasets. The best performance was observed for ResNet50 compared to other two proposed deep models. When compared against its equivalent state-of-the-art 3D CNN model by Zahia et al. [1], proposed models showed considerable improvement in terms of accuracy, sensitivity, specificity, and F-score (Fig 14). Although, proposed pre-trained DL models showed considerably high performance above the state-of-the-art baseline without prior implementation of the proposed Gaussian smoothing and MHN methods, but with further improvement after the implementation of these pre-process operations. The is because, Gaussian smoothing suppresses the interference of Rician noise and other irrelevant information that characterized the used neuroimaging dataset by reducing the discontinuity at the edges and texture of the relevant tissues while MHN method improves the pixel comparability of these tissues.

In summary, developmental dyslexia neural-biomarkers are traceable to tissue densities alterations and activations of both GM and WM in the heterogeneous brain areas with profound effects on a child’s phonological and cognitive behaviours as well as orthographic reading tasks [1,52]. These brain areas, as obtained from MRI-based dyslexia studies, include left and right occipital fusiform gyri, left inferior parietal lobule, left and right supramarginal gyri, cerebellum, precentral gyri, superior and middle temporal gyri, corpus callosum, inferior and superior fasciculus, to mention but a few [10,12,53,54]. The activation patterns of these areas can also be deployed for learning disabilities analysis [55,56].

## Conclusion

Dyslexia is a neuro-developmental cognitive and language disorder with heterogeneous causes and behavioral symptoms. In addition to the MRI dataset used in this study, additional data sources include standardized tests, EEG scans and eye movement tracking [57,58]. The achievement of high classification output using these datasets depends to a large extent on the biological interpretability and comparability of the neural-biomarker features inherent in them.

In this study, we propose a method for improving the comparability and biological interpretability of neuroimage datasets to study dyslexia neural-biomarkers based on proposed MHN and Gaussian smoothing methods. This, we demonstrated by applying three post-processing tasks to already pre-processed MRI datasets using the proposed methods. In this study, a Gaussian filter with an isotropic kernel of size 4mm was employed to smooth the study neuroimaging dataset by removing the noise signals in them prior to intensity normalization. Meanwhile, the proposed MHN method was employed to correct intensity variations between high-quality image and low-quality images. This was achieved by stretching and shifting the histograms of low-quality input images to cover all the available grayscale levels within the range of *ROI*_low_ and *ROI*_high_ of the referenced high-quality image.

Evidence emanated from all results presented show that these two pre-processing tasks become expedient when a study involves the collection of large volumes of different MRI datasets types from multi-sites and multi-parameters scanning centres. This dataset often exhibits large quality variations owing to inconsistent scanner parameter settings. This kind of dataset can limit the reliability of results of post-processing operations such as ROI segmentation, tissue volume estimations, and DL classification, if not appropriately pre-processed before deep feature extraction and classification as the case may require. Meanwhile, the proposed Gaussian smoothing and MHN as well as proposed DL models have been successfully tested on neuroimaging datasets obtained from subjects with age group ranging from 8.7–30 years. It is, therefore, sufficient to state equivocally that age is not a barrier on the performance of the proposed methods.
